# Concurrent treatment with a tumor necrosis factor-alpha inhibitor and veno-venous extracorporeal membrane oxygenation in a post-hematopoietic stem cell transplant patient with idiopathic pneumonia syndrome: a case report

**DOI:** 10.1186/s40560-014-0048-1

**Published:** 2014-08-22

**Authors:** Toshitaka Koinuma, Shin Nunomiya, Masahiko Wada, Kansuke Koyama, Takahiro Suzuki

**Affiliations:** Division of Intensive Care, Department of Anesthesiology and Intensive Care Medicine, Jichi Medical University School of Medicine, 3311-1 Yakushiji, Shimotsuke, Tochigi 329-0498 Japan; Division of Hematology, Department of Medicine, Jichi Medical University School of Medicine, 3311-1 Yakushiji, Shimotsuke, Tochigi 329-0498 Japan

**Keywords:** Idiopathic pneumonia syndrome, Extracorporeal membrane oxygenation, Extracorporeal carbon dioxide removal, Tumor necrosis factor-alpha inhibitor, Etanercept, Hematopoietic stem cell transplantation, Graft-versus-host disease

## Abstract

Idiopathic pneumonia syndrome (IPS) is a fatal non-infectious respiratory complication that develops after hematopoietic stem cell transplantation (HSCT). Because of the poor prognosis of post-HSCT patients with IPS requiring mechanical ventilatory support, performing extracorporeal membrane oxygenation (ECMO) has been regarded as relatively contraindicated in these patients. A tumor necrosis factor-alpha inhibitor, etanercept, has been reported to be a promising treatment option for post-HSCT patients with IPS; however, the phase III clinical trial of etanercept has recently been terminated without definitive conclusion. If post-HSCT patients with IPS really benefit from etanercept, mechanical ventilation (MV)-dependent IPS patients might be worth receiving ECMO treatment in line with the protective lung strategy. We therefore performed veno-venous ECMO (VV-ECMO), which substantially acted as an extracorporeal carbon dioxide removal, on a 56-year-old post-HSCT male with severe MV-dependent IPS due to graft-versus-host disease. Although a serious bleeding complication due to post-HSCT thrombocytopenia occurred, the VV-ECMO was continued for 11 days. The patient successfully entered remission of the IPS and was finally extubated on the 12th MV day. However, the patient soon complained of dyspnea, probably due to cytomegalovirus infection and/or exacerbation of the IPS, and was reintubated after 3 days of extubation. The patient then rapidly developed irreversible type II respiratory failure despite the administration of etanercept and an anti-cytomegalovirus agent and died on the eighth re-MV day. The autopsy findings of the patient revealed diffuse alveolar damage and alveolar hemorrhage, accompanied with bronchitis obliterans in his lungs, as well as whole body cytomegalovirus infection, which were compatible with the clinical diagnosis of the patient. We think that the legitimacy of this treatment strategy is dependent on the overall prognosis of IPS, which is influenced by the complications induced by immunosuppressants and ECMO, especially infections and bleeding.

**Electronic supplementary material:**

The online version of this article (doi:10.1186/s40560-014-0048-1) contains supplementary material, which is available to authorized users.

## Background

Patients with hematological malignancies after hematopoietic stem cell transplantation (HSCT) often suffer from respiratory complications [1], and those who require mechanical ventilation (MV) are known to have a poor prognosis [2]. Idiopathic pneumonia syndrome (IPS), one of the non-infectious respiratory complications that occurs after HSCT, is regarded as an extremely life-threatening condition, and its prevalence is estimated to be 3% to 15% in post-HSCT patients [3]. In particular, MV-dependent post-HSCT patients with IPS have been reported to have a 95% to 100% mortality rate [4-6].

Recently, the effectiveness of a tumor necrosis factor-alpha (TNF-alpha) inhibitor, etanercept, has been aggressively studied to post-HSCT IPS patients [7-9], and thus, MV-dependent IPS patients may also benefit from etanercept treatment [7,8]. Therefore, a lung-protective strategy for MV-dependent IPS patients is considered to be important to avoid volutrauma and/or ventilator-induced lung injury (VILI) until etanercept can exert its effects.

The usefulness of extracorporeal membrane oxygenation (ECMO) for acute respiratory distress syndrome (ARDS) has been reported in recent years [10], but in general, ECMO is considered to be ineffective for patients with malignancies [11,12], and the post-HSCT patients with respiratory complications have been regarded to be relatively contraindicated for ECMO [13]. However, if post-HSCT patients with IPS exhibit an improvement or reversal of their condition by the administration of etanercept, MV-dependent IPS patients might be worth receiving ECMO treatment in line with a lung-protective strategy.

We herein report a post-HSCT case with IPS, in whom we performed veno-venous ECMO (VV-ECMO) because the patient was expected to have a reversal of his condition by the induction treatment with a TNF-alpha inhibitor. To the best of our knowledge, only one very recent case report has been published on an IPS patient who underwent VV-ECMO [14], so our report will be the first on a post-HSCT patient with IPS who was concurrently treated with VV-ECMO and a TNF-alpha inhibitor.

## Case presentation

A 56-year-old male had developed nephrotic syndrome due to primary amyloidosis 12 years earlier and had gone into complete remission following melphalan-prednisone therapy. However, after continuing the melphalan-prednisone therapy for about 10 years, bicytopenia was detected, and he was diagnosed with chronic myelomonocytic leukemia and underwent allogenic bone marrow transplantation (BMT) from a human leukocyte antigen identical sibling donor 11 months prior to his presentation at our hospital. The engraftment of hematopoietic stem cells was observed 24 days after BMT, and acute graft-versus-host disease (GVHD) was not observed while the patient was being treated using cyclosporine as an immunosuppressant. However, the follow-up chimerism analysis revealed the survival of recipient-derived cells, so the cyclosporine was reduced in the hopes of inducing a graft-versus-leukemia (GVL) effect approximately 4 months after the BMT. The cyclosporine was then gradually decreased and discontinued, and the recipient-derived cells almost disappeared due to the GVL effect.

**Figure 1:**
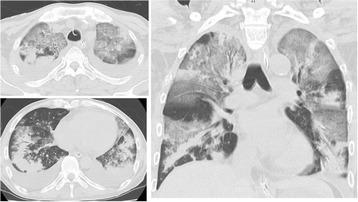
**Computed tomography scans of the patient taken on the first ICU day.** These images show the widespread bilateral peripheral field-dominant patchy consolidations accompanied with diffuse ground glass opacity.

One month before the intensive care unit (ICU) admission, he suffered from an influenza A infection and developed dyspnea with GVHD exacerbation. Cyclosporine re-administration was initiated for the GVHD aggravation, and concurrently, antibiotics, antifungal agents, and antiviral agents were introduced empirically. Despite these therapies, his respiratory symptoms got worse, and he was transferred to the ICU. A computed tomography (CT) examination of his chest revealed bilateral peripheral dominant patchy consolidations accompanied with diffuse ground glass opacity (Figure 1).

**Figure 2:**
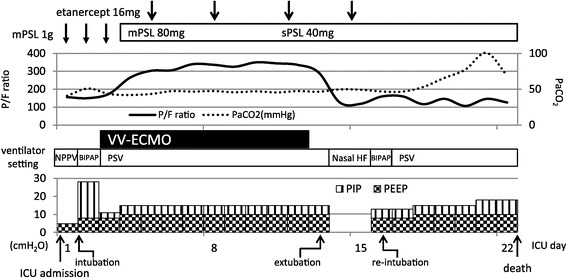
**The ICU course of the patient.** *BIPAP* bilevel positive airway pressure, *F*_*I*_ *O*_*2*_ fraction of inspired oxygen, *mPSL* methylprednisolone, *Nasal HF* nasal high flow system, *NPPV* non-invasive positive pressure ventilation, *PaCO*_*2*_ partial pressure of carbon dioxide, *PaO*_*2*_ partial pressure of oxygen, *PEEP* positive end-expiratory pressure, *PIP* peak inspiratory pressure, *PSV* pressure support ventilation, *sPSL* soluble prednisolone, *VV-ECMO* veno-venous extracorporeal membrane oxygenation.

On admission to the ICU, tachypnea (respiratory rate 36/min) and dyspnea with a low oxygen saturation value by pulse oximetry were observed. We considered that the patient might be suffering from IPS because of the GVHD-related respiratory symptoms, so high-dose methylprednisolone therapy (1 g/day for three days) under non-invasive positive pressure ventilation was initiated from the first ICU day. Unfortunately, that night, the patient developed severe hypoxemia and his consciousness rapidly deteriorated, so he was finally intubated and received MV (Figure 2). The initial MV mode was biphasic positive airway pressure, with an inspiratory fraction of oxygen (F_I_O_2_) of 0.8, a peak inspiratory pressure (PIP) of 28 cmH_2_O, positive end-expiratory pressure (PEEP) of 10 cmH_2_O, respiratory frequency of 15/min and tidal volume of around 6 ml/ideal body weight (BW). However, as his spontaneous breathing force gradually increased, probably due to poor oxygenation and respiratory acidosis (pH 7.159, PaCO_2_ 77.4 mmHg, HCO_3_^−^ 27.8 mmol/l, PaO_2_/F_I_O_2_ 135), along with his poor lung compliance (lung compliance 18 ml/cm H_2_O), his strong inspiratory effort generated a large tidal volume up to 10 ml/ideal BW and an asynchrony with the ventilator was observed. His large tidal volume and asynchrony were sustained irrespective of deep sedation and ventilator setting changes, raising concerns about volutrauma and/or VILI. As he already fulfilled the severity criteria for VV-ECMO due to his Murray's score of 3.25 points on the following day, we decided to introduce ECMO therapy with an expectation of a recovery in his lung injury by the administration of etanercept for the IPS.

After obtaining informed consent, the patient was cannulated with a 19 Fr. cannula for venous drainage into his right femoral vein and a 15 Fr. cannula for oxygen-supplemented blood infusion into his right internal jugular vein. Due to the narrow vascular size of the patient, the catheters we used were smaller in diameter than the general recommendation of VV-ECMO. Because of this small diameter, the ECMO session was initiated with an extracorporeal blood flow of 1.5 to 2 l/min, which was relatively low flow rate, with a sweep gas flow of 2 l/min at 80% oxygen (centrifugal pump, CAPIOX® SP101 PLUS; oxygenator, CAPIOX® LX, TERUMO, Tokyo, Japan), and anticoagulation therapy with unfractionated heparin was initiated to maintain an activated clotting time over 150 s.

**Figure 3:**
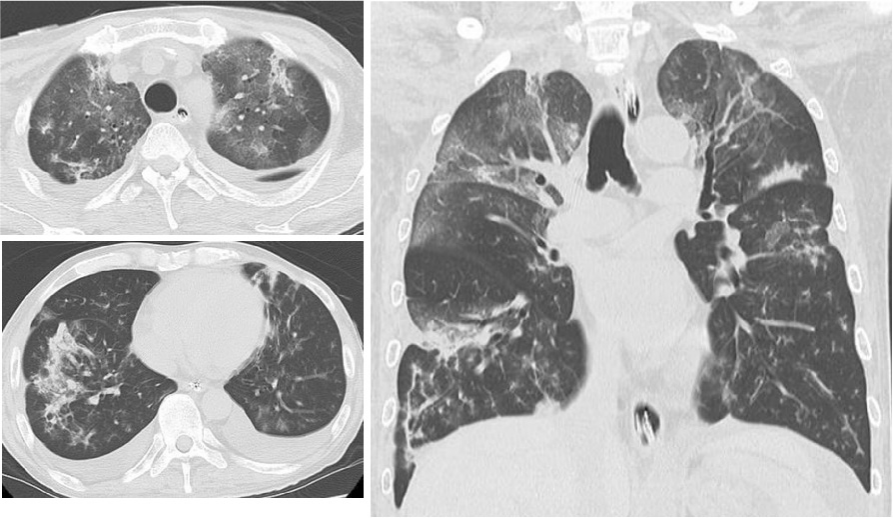
**Computed tomography scans of the patient taken on the 12th ICU day.** These images show the amelioration of the consolidations of the bilateral lung fields.

Soon after the induction of VV-ECMO, the patient was performed bronchoalveolar lavage (BAL) and confirmed to have IPS without any active pulmonary infections based on BAL fluid testing 72 h later, so we administered etanercept subcutaneously at a dose of 16 mg (0.4 mg/kg) twice a week from the fifth ICU day, accompanied by methylprednisolone consolidation therapy (80 mg/day for 7 days and 40 mg/day for the subsequent 7 days). During the ECMO session, he was administered with adequate sedatives and analgesics, and pressure support (PS) ventilation was used with a PEEP of 10 cmH_2_O, PS of 5 cmH_2_O, and F_I_O_2_ of 0.3, so as to maintain lung-rest and prevent alveolar collapse. A few days after the administration of etanercept, his oxygenation was ameliorated up to PaO_2_/F_I_O_2_ 353 mmHg in spite of the low extracorporeal flow rate; nevertheless, his chest X-p showed no sign of improvement of the bilateral consolidations. Therefore, we considered that the patient still had not entered the remission phase of IPS and continued the VV-ECMO, which was substantially acted as ‘extracorporeal carbon dioxide removal (ECCO_2_R)’ , in line with a lung-protective strategy. After further several days, the consolidations in his bilateral lung field almost vanished (Figure 3), so we considered that he had successfully entered remission from the IPS.

On the other hand, he suffered from severe bleeding complications from the vascular access sites used for ECMO, probably due to post-HSCT-related thrombocytopenia, which persisted at less than 2 × 10^4^/μl, and transfusions of platelet concentrate and packed red blood cells were required almost every day during the ECMO session. On the 12th ICU day (the 11th VV-ECMO day), the patient was withdrawn from the ECMO without ventilator setting changes and was successfully extubated on the 13th ICU day. A blood gas analysis performed just before the extubation showed improved oxygenation (pH 7.444, PaCO_2_ 46.5 mm Hg, HCO_3_^−^ 31.2 mmol/l, PaO_2_/F_I_O_2_ 294) under pressure support ventilation with a PEEP of 5 cmH_2_O, PS of 5 cmH_2_O and F_I_O_2_ of 0.4 (Additional file 1: Table S1).

However, the patient experienced a gradually worsening severe cough beginning the day after extubation, and his oxygen saturation was deteriorating despite oxygen therapy, he received a humidified high-flow nasal cannula on the 14th ICU day. As his chest radiography study revealed an exacerbation of the bilateral consolidation, and his blood sample examination revealed seropositivity for the cytomegalovirus antigen, the patient was considered to have developed cytomegalovirus pneumonia and/or an exacerbation of the remitted IPS. Despite the concurrent administration of an anti-cytomegalovirus agent and etanercept, his oxygenation worsened and he required both reintubation and MV support on the 16th ICU day. A few days later, he developed type II respiratory failure, and he died on the 22nd ICU day. The autopsy findings revealed diffuse alveolar damage and alveolar hemorrhage, accompanied with bronchitis obliterans in his lung, as well as whole body cytomegalovirus infection, which were compatible with the clinical diagnosis of the patient.

## Discussion

In 1993, the definition of IPS was proposed as a widespread alveolar injury after HSCT in the absence of active lower respiratory tract infection, which was based upon a BAL examination or lung biopsy, without cardiogenic causes of pulmonary dysfunction [3]. Many factors, such as the use of chemotherapies before BMT, GVHD, and various other factors, including the hematopoietic stem cell donor characteristics, were identified as risk factors for IPS, and it was revealed that TNF-alpha also participates in the onset of IPS [4]. In recent years, a positive impact of a TNF-alpha inhibitor, etanercept, on post-HSCT patients with IPS was reported [7,8], and a phase III clinical trial of etanercept for IPS is conducted [9]. Unfortunately, this trial was not able to show the effectiveness of etanercept for post-HSCT patients with IPS, the definitive conclusion should not be made based on this trial alone because of its inadequate power and small sample size.

According to the report of the Extracorporeal Life Support Organization (ELSO) registry, the results of ECMO for severe respiratory failure in adult patients with malignancy were extremely poor [11]. That study included four adult post-HSCT cases; however, the ELSO registry concluded that it is impossible to make any recommendations because of the small number of cases. The ELSO registry also reported that the result of ECMO for the severe respiratory failure of pediatric post-HSCT patients was extremely poor [12]. Therefore, performing ECMO for post-HSCT patients with severe respiratory failure has been generally considered to be relatively contraindicated by these reports [13]. However, although four adult post-HSCT patients were included in the ELSO report and all had indication of ECMO for pulmonary support [11], there was no mention about their individual causes of respiratory failure, and thus, we have no information about whether these patients suffered from IPS or other diseases. Conversely, a first case report which performed VV-ECMO for an MV-dependent IPS patient was recently published [14]. That paper reported the successful use of VV-ECMO along with conventional treatment for IPS, mainly by glucocorticoid administration, without etanercept. This case report might show the usefulness of the lung rest introduced by ECMO in MV-dependent IPS patients.

Although it is not known whether the results of the CESAR trial [10], which showed the usefulness of VV-ECMO for ARDS, are applicable for other types of severe respiratory failure, such as IPS, we applied the entry criteria of the CESAR trial, especially the Murray's score [15] ≥3 points, to determine whether to introduce VV-ECMO in our case, as it was the only reported case of the successful use of VV-ECMO for IPS [14]. According to the Berlin definition of ARDS [16], however, this case was defined as not the ‘severe type’ but the ‘moderate type’ of ARDS and generally recommended less invasive respiratory supports than ECMO. However, considering the extremely poorer prognosis of MV-dependent IPS patients compared with even ‘severe’ ARDS in the Berlin definition (mortality, 95%–100% [4-6] vs. 45% [16]), we assume that vulnerabilities of MV-dependent IPS patients would be underestimated when we applied this definition to IPS patient population. Moreover, because of our several experiences of IPS patients equivalent to ‘moderate’ ARDS prior to this case, whom were easily complicated VALI in spite of the conventional lung-protective MV setting, we decided to introduce VV-ECMO after due consideration.

Because ECMO is a highly invasive and expensive treatment, it is necessary to judge the indications carefully. Even if using ECMO for IPS is valid to prevent VILI, as was suggested in the aforementioned case report [14], the careful evaluation of the risk-effect ratio with treatment alternatives, which are less invasive and/or less expensive than ECMO, is essential. In particular, it is important to take into consideration the risk of hemorrhagic complications, which may be induced by anticoagulation therapy and serious post-HSCT thrombocytopenia, as was seen in our case.

The reason why the patient exhibited aggravated symptoms soon after the extubation may have included not only a re-exacerbation of IPS but also the cytomegalovirus infection. If the immunosuppression induced by etanercept, along with the high-dose methylprednisolone therapy, is excessive, the concurrent treatment with the induction of etanercept and VV-ECMO for post-HSCT patients with IPS may be considered as a relative contraindication, as is mentioned in the ELSO registry guideline [13].

Although some unresolved problems exist, such as uncertainty of the efficacy of etanercept and VV-ECMO for IPS, the risks of immunosuppression generated by etanercept and other immunosuppressants and the bleeding tendency due to post-HSCT-related thrombocytopenia, we consider that the usefulness of concurrent therapy of immunosuppressants including etanercept and ECMO for IPS deserves an evaluation. However, it should be evaluated by not only the short-term prognosis, such as IPS remission or MV withdrawal, but also the long-term prognosis, such as the overall survival.

## Conclusions

To the best of our knowledge, our case is the second reported post-HSCT patient with IPS on MV who underwent VV-ECMO. In addition, this is the first case treated with concurrent etanercept and VV-ECMO for MV-dependent IPS. We think that the legitimacy of this treatment strategy is dependent on the overall prognosis of IPS, which is influenced by the complications induced by immunosuppressants including etanercept and ECMO, especially infections and bleeding. Further studies on this treatment modality are anticipated in the future.

## Consent

Written informed consent was obtained from the family of the patient for the publication of this case report. A copy of the written consent is available for review by the editor-in-chief of this journal.

## Additional file

Additional file 1: Table S1. The oxygen profile and ECMO setting of the patient during the VV-ECMO session.
