# Supplementary material for: Concurrent treatment with a tumor necrosis factor-alpha inhibitor and veno-venous extracorporeal membrane oxygenation in a post-hematopoietic stem cell transplant patient with idiopathic pneumonia syndrome: a case report
Source: J Intensive Care. 2014 Aug 22;2(1):48. doi: 10.1186/s40560-014-0048-1 (PMC4336281; doi:10.1186/s40560-014-0048-1)
Supplement: Additional file 1: Table S1. — The oxygen profile and ECMO setting of the patient during the VV-ECMO session. [file 40560_2014_48_MOESM1_ESM.docx]

|  |  | **Pre**  **ECMO** | **ECMO**  **Day 1** | **ECMO**  **Day 3** | **ECMO**  **Day 5** | **ECMO**  **Day 7** | **ECMO day 11** | **Post**  **ECMO** |
| --- | --- | --- | --- | --- | --- | --- | --- | --- |
| **ECMO setting** | ECMO flow (l/min)  Sweep gas flow (l/min)  O_2_ ratio of sweep gas | -  -  - | 2.1  2.0  0.8 | 1.8  2.0  0.8 | 2.0  2.0  0.8 | 2.0  2.0  0.8 | 2.1  2.0  0.8 | -  -  - |
| **Circulatory profile** | Cardiac output (l/min)*  Hemoglobin (g/dl) | N.E.  8.1 | 5.5  8.8 | 5.1  8.3 | 4.6  8.2 | 5.3  10.9 | 4.5  9.2 | 7.0  10.2 |
| **Ventilator setting** | F_I_O_2_  PEEP (cm H_2_O)  Minutes volume (l/min)  Tidal volume (ml/kg) | 0.8  10  6.4  6.8 | 0.5  8  1.7  5.9 | 0.4  10  3.5  6.6 | 0.3  10  1.9  4.6 | 0.3  10  2.1  5.3 | 0.3  10  2.3  5.1 | 0.4  5  9.3  9.5 |
| **Blood gas analysis** | pH  PaCO_2_ (mm Hg)  PaO_2_ (mm Hg)  SaO_2_ (%)  HCO_3_^-^ (mmol/l) | 7.159  77.4  107.9  97  27.8 | 7.344  46.7  61.2  92.1  25.6 | 7.445  41.9  105.8  97.5  28.1 | 7.394  47.3  91.8  96.4  28.2 | 7.430  43.6  89.8  96.6  28.3 | 7.432  47.4  100.8  97.2  30.9 | 7.444  46.5  117.4  98.2  31.2 |

**Additional file 1: Table S1 The oxygen profile and ECMO setting of the patient during the VV-ECMO session**

*ECMO* extracorporeal membrane oxygenation, *F_I_O_2_* fraction of inspired oxygen, *HCO_3_^-^* bicarbonate ion, *N.E.* not examined, *PaCO_2_* partial pressure of carbon dioxide, *PaO_2_* partial pressure of oxygen, *PEEP* positive end-expiratory pressure, *VV-ECMO* veno-venous extracorporeal membrane oxygenation. *The cardiac output of the patient was measured using FlowTrac^TM^ (Edwards Lifesciences Japan, Tokyo, Japan).
